# A New Onset of Vocal Tic in an Adult Lady Secondary to Brain Metastasis

**DOI:** 10.7759/cureus.55051

**Published:** 2024-02-27

**Authors:** Khawlah Fairaq, Moayyad Alsalem

**Affiliations:** 1 Psychiatry, Department of Medicine, King Abdulaziz Medical City, Ministry of the National Guard - Health Affairs, Jeddah, SAU; 2 Psychiatry, King Abdullah International Medical Research Center, Jeddah, SAU; 3 Psychiatry, College of Medicine, King Saud Bin Abdulaziz University for Health Sciences, Jeddah, SAU

**Keywords:** motor tic, primary or brain metastasis, tic diosrder, adulthood onset, vocal tic

## Abstract

Tics are neuropsychiatric events characterized by brief, rapid motor movements or vocalizations in response to irresistible premonitory urges. They start in childhood and may or may not persist in adulthood. Adulthood-onset tics are usually an extinction of undiagnosed tics in childhood. Secondary tic disorders may be due to brain tumors, medications, or neurological disorders.

In this article, we are describing a woman in her 60s who presented with a new-onset vocal tic a few years after being diagnosed with gluteal sarcoma that metastasized to the lung and brain, involving the left temporal and right occipital lobes.

This case is similar to previously reported case reports of secondary tic disorders, although the type of tic, the brain region involved, and the primary tumors are different.

## Introduction

Tics are neuropsychiatric events characterized by brief, rapid motor movements or vocalizations that occur in response to irresistible, premonitory urges [1]. These involuntary actions can take three forms: motor only (physical movements), phonic only (vocalizations), or a combination of both.

Tics commonly emerge in childhood, between the ages of five and six. In a typical course, they tend to improve significantly or even fully by adolescence or adulthood [2].

While the diagnostic criteria specify that tics must typically begin before 18 years old, several studies have documented cases of tic disorders starting in adulthood. These are categorized as primary or secondary. Primary adult-onset tic disorder often involves the resurfacing of childhood tics that initially subsided and later reappear [3]. On the other hand, secondary adult-onset tic disorder is caused by external factors such as medications, brain injuries, or other neurological conditions. These tics usually resolve once the underlying cause is addressed.

In adults, combined motor and phonic tics are the most common type, followed by motor tics alone. Phonic tics alone are the least frequent [4].

This article presents the case of an adult woman who developed vocal tics for the first time at the age of 60.

## Case presentation

**Figure 1:**
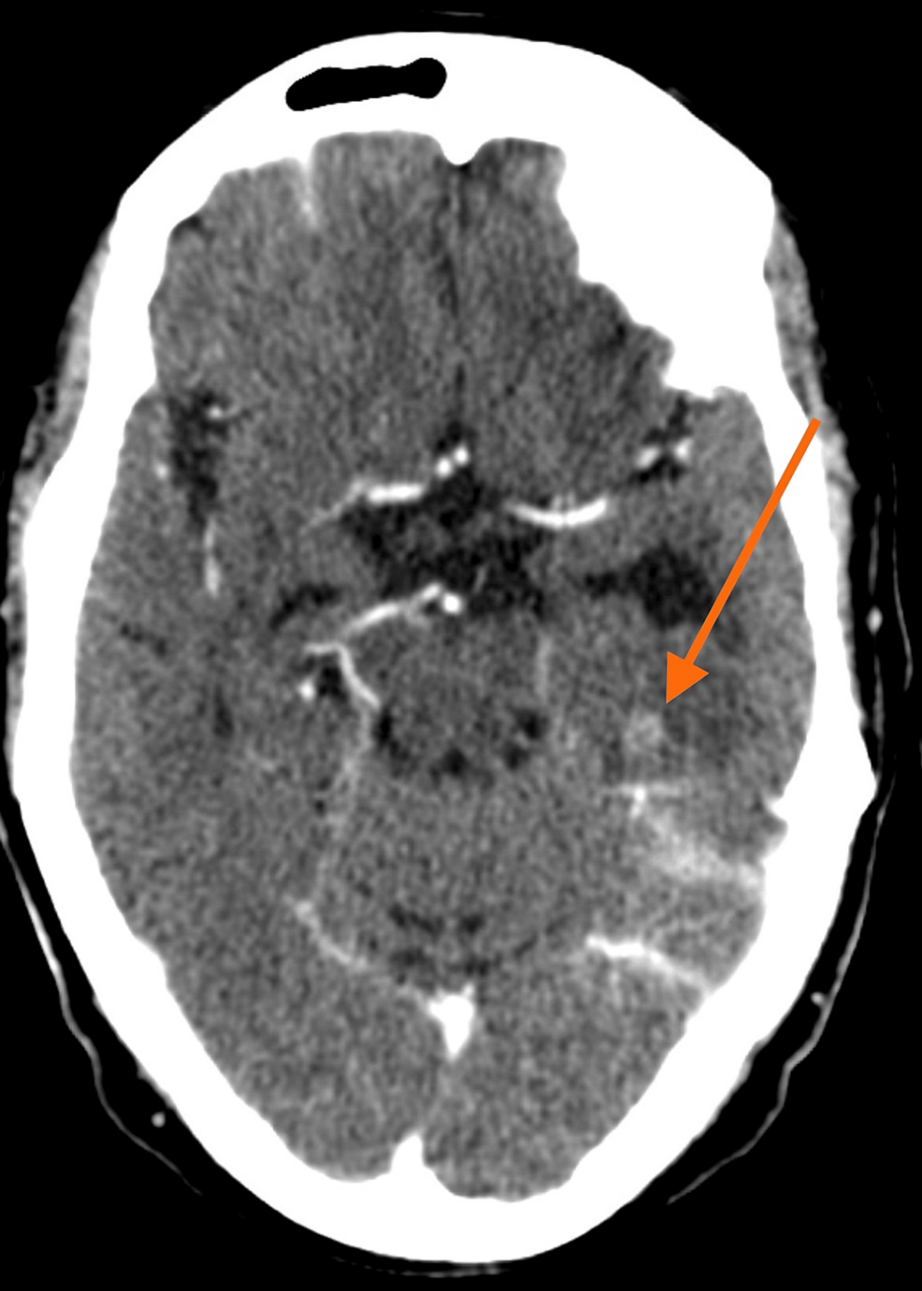
Brain CT shows metastasis in the left temporal lobe.

 A woman in her 60s with no prior psychiatric history was referred to the Department of Psychiatry from Oncology for evaluation of new-onset involuntary vocalizations. Her medical history was significant for a diagnosis of gluteal sarcoma in the middle 50s, with subsequent metastasis to the lung and brain three years later. She had received multiple rounds of chemotherapy and radiotherapy and underwent regular brain CT scans (Figure 1), which demonstrated stable lesions in the left temporal and right occipital lobes.

Three months prior to the presentation, she reported suddenly experiencing involuntary vocalizations characterized by loud, wordless screams. These episodes occurred every two to five minutes throughout the day in both public and private settings, regardless of the presence of others. She denied any premonitory urges, obsessions, hallucinations, or emotional triggers associated with the vocalizations. Notably, she described a complete absence of awareness or cognitive processing prior to the screaming and felt surprised each time it happened. Despite the involuntary nature, she expressed a lack of urge to resist the vocalizations and simply returned to her previous activity afterwards. The episodes did not provide any relief and ceased only during sleep or prayer.

She denied any other abnormal voices, involuntary speech, or behavior, and she had no prior history of involuntary movements. The vocalizations significantly impacted her quality of life, limiting her social interactions and causing distress to herself and her family. A comprehensive psychiatric evaluation revealed no evidence of mood, anxiety, or psychotic symptoms.

The initial differential diagnosis for this patient, given her age and medical history, included psychosis secondary to a medical condition (brain metastasis). Although the presence of stable brain lesions warrants consideration, the absence of typical psychotic symptoms, such as disorganized speech, delusions, or hallucinations, makes this diagnosis less likely. Another differential diagnosis is an obsessive-compulsive disorder with screaming as a compulsion; her lack of pre-screaming intrusive thoughts or urges and post-screaming relief makes this diagnosis improbable.

Following these considerations, we focused on evaluating the vocalizations as an isolated symptom with significant functional impairment. This presentation most closely resembles a vocal tic disorder, albeit with the unusual feature of adult-onset.

While the patient lacks a childhood history of tics, adult-onset vocal tic disorder, unspecified, appears as the most appropriate diagnosis at this stage. Further investigations, including neuroimaging with functional correlates and detailed neuropsychological evaluation, may help clarify the specific etiology and inform treatment decisions.

Consistent with the working diagnosis of adult-onset vocal tic disorder unspecified, a two-pronged approach was initiated. Pharmacological intervention commenced with haloperidol, an FDA-approved medication for tic disorders, starting at a low dose of 2.5 mg orally once daily. This dosage choice balances potential efficacy with minimizing side-effect risks. In addition, a referral to the Neurology Department was made for further evaluation to rule out alternative etiologies such as focal seizures.

Furthermore, psychoeducation was provided to the patient and her family. This covered information about the nature and management of vocal tic disorders, including what to expect from the treatment plan and potential coping strategies.

Unfortunately, the patient's clinical course took a significant turn. One month after treatment initiation, she developed shortness of breath and was admitted to the oncology ward. Sadly, she passed away shortly thereafter.

Given these circumstances, we were unable to fully assess her response to the treatment plan or conduct further investigations and adjustments as necessary. Therefore, long-term management and follow-up for her vocal tic disorder were not achieved.

## Discussion

Adult-onset tic disorders are relatively uncommon, often arising secondary to various brain insults. It is important to review the pathophysiologic hypotheses of primary tic disorder in order to understand the possible mechanisms underlining secondary tic disorder.

There are two brain areas that are mostly hypothesized to play a role in tic disorder. The first one is the cortex. This is supported by the frequent comorbidities of neuropsychiatric disorders associated with tic disorder and the presence of preceding sensory premonitory urges, although urges are absent in our case. As well as volumetric magnetic resonance imaging (MRI) studies reporting changes in certain cortical areas, including prefrontal, frontal, sensorimotor, cingulate, parietal-occipital, and temporal areas [5]. It is worth mentioning that the brain metastasis in our patient involved the occipital and temporal areas, which might explain her presentation. The second hypothesized area is the basal ganglia; this finding was also consistent in secondary tic disorder [5,6]. Yet, basal ganglia is spared in our case.

This phenomenon of adult-onset tic has been documented in several case reports, with our case likely falling under this category due to the presence of brain metastases.

Several case studies support this connection. In 2009, Luat et al. described an 11-year-old boy with a large temporal-lobe oligodendroglioma extending to the basal ganglia. He presented with a complex picture of attention deficit hyperactivity disorder (ADHD), obsessive-compulsive disorder (OCD), stimulant-induced tic disorder, and seizures, significantly improving after tumor resection [7]. Similarly, Iwasaki et al. reported an adult patient with right trigeminal neuralgia and ipsilateral facial spasms, both considered convulsive tics secondary to a brain tumor. Subtotal resection for nerve decompression resulted in complete symptom resolution [8]. Gomis et al. further suggest caudate nucleus stroke as another potential cause of adult-onset tics [9].

Similarities in the clinical presentation are found between our case and previously reported cases of secondary tic in terms of lacking associated features, such as urge, distractibility, suppressibility, and suggestibility, in contrast to primary tic disorder [6].

The management of adult-onset tic disorders hinges on differentiating between primary and secondary origins. While addressing the underlying cause is crucial, several studies report positive responses to common tic disorder medications. These include antipsychotics (primarily haloperidol and pimozide), beta-blockers, tetrabenazine, and benzodiazepines, with varying effectiveness across individuals [4].

## Conclusions

The potential for significant distress and social isolation in adult-onset tic disorder highlights the importance of considering this diagnosis even in later life. As demonstrated in our case, where the patient avoided public settings due to embarrassment, tics can severely impact the quality of life. Therefore, we recommend a thorough history-taking and physical examination for early detection of new, developing neurological signs, such as tic, in patients with cancer. As well as investigation for potential underlying causes to inform appropriate management strategies. Taking early action on these aspects can positively impact patients' well-being and social engagement.
